# Soy Consumption, but Not Dairy Consumption, Is Inversely Associated with Fatty Acid Desaturase Activity in Young Adults

**DOI:** 10.3390/nu13082817

**Published:** 2021-08-17

**Authors:** Melissa Gonzalez-Soto, Salma A Abdelmagid, David W.L. Ma, Ahmed El-Sohemy, David M Mutch

**Affiliations:** 1 Department of Human Health and Nutritional Sciences, University of Guelph, Guelph, ON N1G 2W1, Canada; mgonza08@uoguelph.ca (M.G.-S.); salma.abdelmagid@gmail.com (S.A.A.); davidma@uoguelph.ca (D.W.L.M.); 2 Department of Nutritional Sciences, University of Toronto, Toronto, ON M5S 1A8, Canada; a.el.sohemy@utoronto.ca

**Keywords:** humans, fluid milk, dairy, soy beverage, plant-based beverage, LC-PUFA, delta-6 desaturase

## Abstract

Past research using hepatic rat microsomes showed that soy protein suppressed delta-6 desaturase activity (D6D) compared to casein (a dairy protein). The effects of soy and dairy on desaturase pathway activity in humans remain poorly investigated. The objective of this analysis was to investigate the association between soy and dairy consumption with plasma fatty acids and estimate the desaturase pathway activity in a multiethnic Canadian population of young adults. We analyzed data from men (*n* = 319) and women (*n* = 764) previously collected for the Toronto Nutrigenomics and Health Study. Food frequency questionnaires and plasma fatty acids were assessed. Relationships between soy and dairy beverages and food consumption with estimated desaturase activities were assessed by regression models and by grouping participants according to beverage and food intake data. Weak inverse associations (*p* ≤ 0.05) were found between soy consumption and the overall desaturation pathway activity, specifically D6D activity. When participants were grouped based on soy and dairy consumption habits, omega-6 LC-PUFAs, as well as various estimates of the desaturase pathway activity, were significantly lower in individuals consuming soy (with or without dairy) compared to individuals consuming only fluid milk and dairy products. In conclusion, soy consumption, not dairy consumption, appears to suppress desaturase pathway activity.

## 1. Introduction

Bovine milk is a rich source of nutrients such as protein, fat, vitamins and minerals [1]. Despite this, fluid milk intake has declined among North Americans over the past decade, while the consumption of dairy foods (e.g., cheese and yogurt) has grown [2,3]. This can be due, in part, to the wide availability and popularity of plant-based alternatives to fluid milk, such as soy beverage [2,4]. Additionally, changes in consumer preferences are often attributed to general concerns about the saturated fatty acid content of milk and the association between these fats and cardiovascular risk, among others [2,5]. However, recent meta-analyses reported a neutral association between fluid milk consumption and clinical outcomes such as stroke, coronary heart disease and type 2 diabetes [6,7,8]. Moreover, soy protein intake is reported to improve overall lipid profiles through different pathways [9]. While the hypocholesterolemic and hypotriglyceridemic effects of increased soy consumption are well documented, evidence suggests that soy protein may inhibit delta-6 desaturase (D6D) activity, a key enzyme involved in long-chain polyunsaturated fatty acid (LC-PUFA) endogenous synthesis.

LC-PUFA are bioactive molecules that constitute cell membrane phospholipids, serve as important signalling molecules, and are precursors for the production of bioactive lipid mediators [10]. LC-PUFA are obtained from dietary sources or synthesized endogenously in the human body from essential fatty acids (e.g., linoleic acid, LA; α-linolenic acid, ALA). However, this conversion occurs to a limited extent, with tracer studies indicating that <8% and <1% of ALA is converted to eicosapentaenoic acid (EPA) and docosahexaenoic acid (DHA), respectively [11,12]. This conversion pathway involves the desaturation and elongation of essential fatty acids by specific enzymes. Specifically, D6D and the delta-5 desaturase (D5D) insert double bonds at specific positions in a fatty acid chain, while the elongation of the very-long-chain fatty acids protein 2 (ELOVL2) and 5 (ELOVL5) extend a fatty acid chain by 2 carbons (as reviewed in [13]). These enzymes are influenced by numerous dietary factors, such as macronutrients, micronutrients and polyphenols [13], as well as by non-dietary factors including sex, BMI, age, smoking and alcohol consumption [14].

Past studies in rats showed that soy protein consumption impaired D6D activity compared to casein [15,16]. Specifically, it was shown that D6D activity, assessed by the conversion of radiolabeled LA into γ-linolenic acid (GLA), was reduced in hepatic microsomes isolated from rats consuming a soy-supplemented diet compared to a casein-supplemented diet. While intriguing, the translatability of these in vitro results to humans is unclear due to the fact that microsomes are cellular artifacts enriched in endoplasmic reticulum proteins, but absent of cytoplasmic proteins [17].

The primary objective of this analysis was to determine if the consumption of soy beverage and fluid milk was associated with plasma omega-6 and omega-3 PUFA levels and the estimated desaturase activities in a cohort of young healthy adults. As a secondary objective, we conducted a broader analysis to evaluate if the intake of dairy foods in the presence or absence of soy foods was associated with omega-6 and omega-3 PUFA levels, and estimated desaturase activities.

## 2. Materials and Methods

### 2.1. Study Population

**Figure 1:**
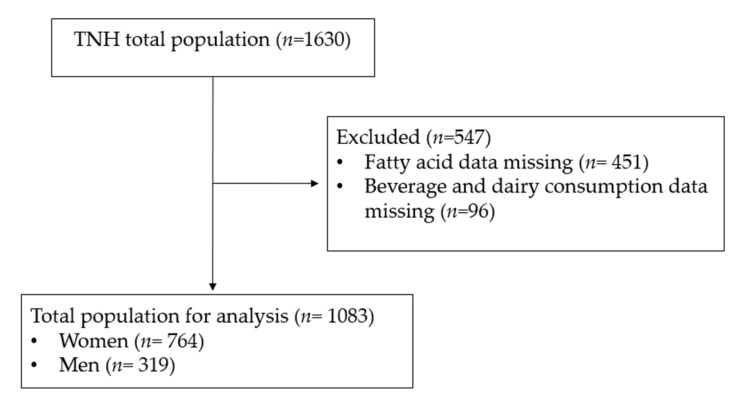
CONSORT diagram. Subject inclusion and exclusion criteria.

The present study used data previously collected from the cross-sectional Toronto Nutrigenomics and Health (TNH) study. Participants (*n* = 1630) between the ages of 20 and 29 years were recruited from the University of Toronto between 2004 and 2010. Individuals were excluded from the analysis if fatty acid data (*n* = 451) and soy/dairy food consumption data (*n* = 96) were missing (Figure 1).

### 2.2. Dietary Intake Assessment

The Toronto-modified Willett food frequency questionnaire (FFQ) was used at one timepoint to collect information about diet and beverage intake over the preceding month. The original FFQ was modified to include foods that contributed to the glycemic index of the diet and to assess the consumption of caffeine, as previously described [18]. For the purpose of the present analysis, FFQ data related to the total caloric intake (kcal/d), total omega-3 intake (calculated by summing dietary ALA + EPA + 22:5*n-*3 (DPA) + DHA), total omega-6 intake (calculated by summing dietary LA + AA) intake, fish oil supplement use, and consumption of fluid milk (skimmed milk, 1% milk, 2% milk, whole milk and flavored milk), soy beverage, as well as the total soy and total dairy foods, were considered. Total soy comprised tofu, soybean and soy beverage servings, while total dairy comprised fluid milk (skimmed milk, 1% milk, 2% milk, whole milk and flavored milk), cream, ice cream, yogurt, cheese, and butter servings. For simplicity, the intake of soy beverage, fluid milk, soy foods, and dairy foods was converted into servings per month from the Willett FFQ.

### 2.3. Fluid Milk versus Soy Beverage Analysis

For our primary analysis, study participants were divided into four different groups depending on their fluid milk and soy beverage consumption habits (i.e., servings per month). Individuals that consumed 1 or more servings of fluid milk (equivalent to 8 oz or 240 mL) per month and no servings of soy beverage were designated the “Milk” group (*n* = 671; mean intake ± standard error = 30.6 ± 1.1 servings). Individuals that consumed 1 or more servings of soy beverage (equivalent to 8 oz or 240 mL) per month and no fluid milk servings were designated the “Soy” group (*n* = 71; mean intake ± standard error = 22.5 ± 3.1 servings). Individuals that consumed both fluid milk and soy beverage (i.e., at least 1 serving per month of each) were designated the “Both” group (*n* = 269; mean intake fluid milk ± standard error = 24.8 ± 1.6 servings; mean intake ± standard error soy beverage = 11.9 ± 0.9 servings), while individuals that consumed less than 1 serving per month of fluid milk and soy beverage were designated the “No consumption” group (*n* = 72).

### 2.4. Total Dairy and Total Soy Analysis

For our secondary analysis, study participants were divided into four different groups depending on their total dairy and total soy food consumption habits (i.e., servings per month). The “Dairy” group (*n* = 401) comprised individuals who consumed 1 or more servings per month of any type of dairy (mean intake ± standard error = 72.2 ± 2.2 servings). The “Both” group (*n* = 663) comprised individuals who consumed 1 or more servings per month of any type of dairy and 1 or more servings per month of any type of soy food (mean intake dairy ± standard error = 62.1 ± 1.7 servings; mean intake soy ± standard error = 15.1 ± 0.9 servings). The number of individuals that consumed no dairy foods and only soy foods (*n* = 15), or neither dairy foods nor soy foods (*n* = 4), were too low to consider in our analyses. Therefore, our secondary analysis only examined the “Dairy” and “Both” groups.

### 2.5. Anthropometric and Clinical Measurements

Anthropometric measurements were obtained from all study participants by a trained professional, as previously described [18]. Body mass index (BMI) was calculated using the weight (kg) and height (m) of the participant. Blood samples were obtained from participants following a 12-h overnight fast. Plasma was extracted and used to analyze fatty acids and biochemical markers such as glucose, insulin, cholesterol (total, HDL, and LDL), triglycerides and high sensitivity C-reactive protein (hs-CRP).

### 2.6. Plasma Fatty Acid Analysis

Total fatty acids were extracted from plasma samples collected after an overnight fast and analyzed by gas chromatography, as previously described [19]. The ratio C17:0 was used as an internal standard to measure fatty acid concentration. Relative fatty acid data, expressed as a percentage of total fatty acid content, were used to estimate fatty acid desaturase and elongase activities using product-to-precursor ratios. Only PUFA data is reported in the present manuscript. The ratios of GLA/LA (18:3*n*-6/18:2*n*-6), arachidonic acid (AA)/di-homo-ɣ-linolenic acid (DGLA) (20:4*n*-6/20:3*n*-6), DGLA/GLA and adrenic acid/AA (22:4*n*-6/20:4*n*-6) were used to estimate D6D, D5D, ELOVL5 and ELOVL2 activities, respectively. Furthermore, the AA/LA and the EPA/ALA ratios were used to estimate the overall activity of the desaturation pathway.

### 2.7. Statistical Analysis

All analyses were conducted using JMP statistical software V14.0.0 (SAS Institute, Cary, NC, USA). The Shapiro–Wilk test was used to assess covariates for normality. We first ran exploratory correlations between servings per month for fluid milk, soy beverage, total soy and total dairy with estimated enzyme activities using a Pearson’s correlation test. Multivariate linear regression was then used to model the relationships between fluid milk, soy beverage, total soy and total dairy servings per month and estimated enzyme activities to account for the following variables known to influence fatty acid desaturation: sex, BMI, waist circumference, age, ethnicity, total caloric intake and fish oil supplementation [14]. Plasma ALA and LA concentrations (as measured by gas chromatography) were included in our models to account for potential differences in essential fatty acid intake. For subgroup analyses, differences in fatty acid levels and ratios were assessed using an ANCOVA with a Tukey post hoc analysis for our primary analysis (fluid milk vs. soy beverage) and a Student’s *t*-test for the secondary analysis (total dairy vs. total dairy/total soy). Models for fatty acid sub-group analyses included the following covariates: sex, BMI, age, waist circumference, ethnicity, total caloric intake, and omega-3 supplementation. Models for fatty acid ratio sub-group analyses included the aforementioned covariates, as well as plasma ALA, LA and DHA levels (μg/mL). Data are reported as mean ± standard deviation. A *p*-value ≤ 0.05 was considered statistically significant.

## 3. Results

### 3.1. Correlations between Soy and Dairy Beverage and Food Consumption and Desaturation Indices

**Figure 2:**
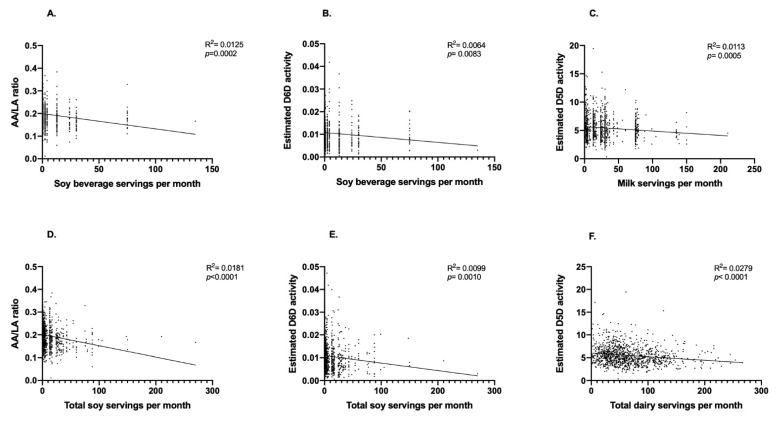
Relationships between soy beverage, fluid milk, total soy and total dairy intake with estimated desaturase activities. Pearson’s correlation was used to initially explore the relationships between soy beverage, fluid milk, total soy and total dairy intake with estimated desaturase activities. (**A**) Monthly servings of soy beverage and the AA/LA ratio, (**B**) monthly servings of soy beverage and estimated D6D activity, (**C**) monthly servings of fluid milk and estimated D5D activity, (**D**) monthly servings of total soy and the AA/LA ratio, (**E**) monthly servings total soy and estimated D6D activity, and (**F**) monthly servings of total dairy and estimated D5D activity; *n* = 1083 participants.

A total of 1083 participants (*n* = 764 women and *n* = 319 men) were included in the present analysis (Figure 1). We first performed exploratory analyses to determine if the consumption of soy beverage, fluid milk, total soy and total dairy intake was correlated with estimated enzyme activities. Weak but statistically significant inverse correlations were observed between soy beverage intake and the AA/LA ratio (R^2^ = 0.0125; *p* = 0.0002; Figure 2A) and estimated D6D activity (R^2^ = 0.0064; *p* = 0.0083; Figure 2B). No significant relationships were found between soy beverage intake and estimated D5D, ELOVL2 or ELOVL5 activities. After adjusting for confounding variables in a multivariate model, the relationship between soy beverage consumption and AA/LA estimated activity, as well as with D6D, remained statistically significant (*p* ≤ 0.05). A weak inverse correlation was found between fluid milk intake and estimated D5D activity (R^2^ = 0.0113; *p* = 0.0005; Figure 2C); this relationship remained significant after adjusting for confounding variables (*p* ≤ 0.05). Weak but statistically significant positive correlations were also found between fluid milk intake and estimated D6D activity (R^2^ = 0.0053; *p* = 0.0163) and the EPA/ALA ratio (R^2^ = 0.0054; *p* = 0.0153); however, these relationships were lost in a multivariate model accounting for covariates (*p* > 0.05). No associations were found with fluid milk intake and estimated elongase activities. Similar to soy beverage, significant inverse correlations between total soy and the AA/LA ratio (R^2^ = 0.0181; *p* < 0.0001; Figure 2D) and estimated D6D activity (R^2^ = 0.0099; *p* = 0.0010; Figure 2E) were found. Both relationships remained significant after accounting for confounding variables (*p* ≤ 0.05). No other associations were found with total soy intake and estimated enzyme activities. Similar to fluid milk, an inverse correlation was also found between total dairy intake and estimated D5D activity (R^2^ = 0.0279; *p* = <0.0001; Figure 2F), which remained significant after adjusting for confounding variables (*p* ≤ 0.05). A positive correlation was also observed between total dairy intake and estimated D6D activity (R^2^ = 0.0057; *p* = 0.0132); however, this relationship was lost after accounting for confounding variables (*p* = 0.2247). No associations were found with total dairy intake and estimated elongase activities.

### 3.2. Influence of Fluid Milk and Soy Beverage Consumption on Plasma Fatty Acids and Related Ratios

Considering the weak but statistically significant correlations identified in our exploratory analyses, we next conducted a primary analysis to investigate if relative plasma fatty acids and estimated enzyme activities differed in individuals categorized based on their fluid milk and/or soy beverage consumption habits. Small significant differences in age (*p* = 0.0468), BMI (*p* = 0.0058) and total caloric intake (*p* = 0.0127), were observed when comparing beverage consumption groups (Table 1). The dietary omega-6/omega-3 ratio determined from FFQ data was borderline significant between groups (*p* = 0.0539). ALA (*p* = 0.0254) and LA (*p* = 0.0036) plasma levels (µg/mL), as determined by gas chromatography, differed between groups (Table 1), where subjects in the “Soy” group had the highest LA plasma levels compared to the other groups. No pairwise differences in ALA levels were identified with the post hoc analysis. The plasma LA/ALA ratio was borderline significant (*p* = 0.0451), although no pairwise post hoc differences were observed.

The relative (%) plasma levels of ALA and omega-6 PUFA differed significantly between groups (Table 2). Individuals consuming soy beverage with or without fluid milk (“Soy” and “Both” groups) had higher plasma levels of ALA (0.83 ± 0.27% and 0.79 ± 0.22%, respectively) compared to individuals consuming only fluid milk (0.72 ± 0.26%), as well as higher levels of LA (34.17 ± 3.65% and 33.42 ± 3.87%, respectively), compared to individuals consuming only fluid milk (31.81 ± 3.94%) and individuals without beverage consumption (31.78 ± 5.09%). ALA and LA levels were similar between individuals in the “Milk” and “No Consumption” groups, and between individuals in the “Soy” and “Both” groups. No significant differences were observed with EPA between the groups. Individuals consuming soy beverage had the lowest DHA levels (1.42 ± 0.54%) compared to the rest of the groups. Plasma levels of GLA were lower in individuals in the “Both” group (0.30 ± 0.16%) compared to those in the “Milk” group (0.34 ± 0.16%), while DGLA and AA levels were similar between individuals in the “Soy” and “Both” groups, and were significantly lower than the levels observed in individuals in the “Milk” and “No consumption” groups.

We next examined the estimates for the overall desaturation pathway activity using both omega-3 and omega-6 PUFA. The AA/LA ratio was significantly lower in individuals in the “Both” group compared to individuals in the “Milk” and “No Consumption” groups, suggesting a general suppression in desaturation pathway activity. This same ratio was also marginally lower in the “Soy” group compared to the “Milk” and “No Consumption” groups. Since we were unable to consistently detect all *n*-3 PUFA intermediates in the desaturation pathway, we used LA, GLA, DGLA, and AA to estimate individual desaturase and elongase activities. The estimated D6D activity was significantly lower in individuals in the “Both” group compared to individuals in the “Milk” and “No Consumption” groups, which aligned with results concerning the overall pathway (AA/LA). No differences were observed in the estimated D5D, ELOVL2, and ELOVL5 activities. Collectively, these observations suggest that soy beverage consumption but not milk consumption may be associated with reduced desaturation pathway activity.

### 3.3. Fatty Acid Levels and Desaturation Indices by Total Dairy and Soy Consumption Groups

In a secondary analysis, we investigated the relative plasma fatty acids and estimated enzyme activities in individuals categorized based on total dairy and total soy intake. Similar to the observations from our primary analysis, significant differences were found in BMI (*p* < 0.0001), hs-CRP (*p* = 0.0269), and LDL (*p* = 0.0082) values between groups, where individuals consuming only dairy had higher values compared to individuals consuming both dairy and soy (Table 3). HDL levels were also different between groups, where individuals in the “Both” group had higher levels in plasma (*p* = 0.0192). No significant difference between the two groups was observed for the dietary omega-6/omega-3 ratio based on FFQ data. ALA (*p* = 0.0057) and LA (*p* = 0.0003) plasma levels (µg/mL) were higher in individuals in the “Both” group compared to those in the “Dairy” group, while the LA/ALA ratio was significantly higher in individuals in the “Dairy” group (*p* = 0.0301).

The relative (%) plasma fatty acids also differed between individuals in these two groups (Table 4). Individuals in the “Both” group had significantly higher levels of ALA, LA, and DHA compared to individuals in the “Dairy” group. By contrast, GLA, DGLA and AA were all lower in individuals in the “Both” group compared to the “Dairy” group. No differences in EPA levels were observed between the two groups (Table 4).

## 4. Discussion

In the present study, we report that soy intake, irrespective of dairy consumption, is inversely associated with the overall desaturase pathway activity, as well as with estimated D6D activity. Our initial exploratory correlation analyses showed that soy consumption is inversely associated with desaturase activity. These observations were supported by both the primary analysis comparing soy beverage and fluid milk intake, as well as the secondary analysis comparing the total dairy intake with and without the consumption of soy foods. We also observed that fluid milk intake, as well as total dairy intake, was inversely associated with estimated D5D activity. While potentially intriguing, this relationship was not found in any of our subgroup analyses and therefore not considered further.

A strength of our primary analysis was that we could create four distinct groups of individuals based on their fluid milk and soy beverage consumption habits: fluid milk only, soy beverage only, both, and neither. This showed that the consumption of soy beverage, but not fluid milk, was associated with a reduction in estimated D6D activity. This was particularly evident when comparing the two largest subgroups (“Milk” vs. “Both”). We recognized that fluid milk is not the only source of dairy in the diet, and that there are other sources of soy protein as well; therefore, our secondary analysis compared individuals consuming any type of dairy with and without any type of soy. This secondary analysis aligned with our initial findings, and also showed that the inclusion of soy foods in a diet containing dairy foods is associated with a reduction in estimated D6D activity. Collectively, these findings may have important implications for public health nutrition, in particular for individuals who consume plant-based alternatives to dairy and have diets low in omega-3 LC-PUFA.

To the best of our knowledge, this is the first cross-sectional cohort study to assess the relationship between soy intake and estimated desaturase activities in humans. Past animal studies suggested that soy protein consumption decreased LC-PUFA synthesis by inhibiting D6D activity. Brandsch et al. [20] fed rats diets containing either soy or casein as a source of protein. Higher microsomal LA levels concomitant with lower microsomal levels of DGLA, AA and a lower AA/LA ratio, were observed in rats fed soy compared to rats fed casein, suggesting a suppression in the activity of the LC-PUFA desaturation pathway. In two independent studies, Ikeda et al. [16] and Koba et al. [15] both reported suppressed microsomal D6D activity (measured using radiolabeled fatty acids) and lower levels of LC-PUFAs such as EPA and AA in hepatic microsomes isolated from rats who were fed soy compared to casein. Together, this evidence suggested that soy protein consumption decreased LC-PUFA synthesis through a reduction in D6D activity compared to casein consumption.

Although comparative research regarding the effects of soy protein and dairy (more specifically casein) on lipid metabolism exists, the effects of soy and dairy on desaturation pathway activity in humans remain poorly investigated. To the best of our knowledge, only one study examined the impact of soy protein and casein supplementation on plasma fatty acids in humans. Specifically, Gooderham et al. [21] studied the effects of a soy protein isolate and a casein supplement on plasma phospholipid fatty acid profiles in 20 young male adults randomly allocated to each treatment (60 g/d for 28 days). Plasma levels of AA showed lower trends in subjects supplemented with soy protein isolate compared to those given the calcium caseinate supplement, but no significant difference was seen in the estimated overall desaturation pathway activity. It is difficult to directly compare the outcomes from a short-term intervention with a small sample size (*n* = 10 per supplement group) with our cross-sectional cohort analysis, since the first is assessing supplementation while the second is assessing general dietary patterns. Nevertheless, the trend for the reduction in AA levels observed during the wash-out period after soy supplementation in the intervention trial may suggest a longer-lasting regulation of the desaturation pathway that aligns with our findings.

Although the present investigation provides no mechanistic insight, the suppressive effects of soy on desaturase activity may be mediated at the transcriptional level via sterol regulatory element binding protein-1 (SREPB-1). Indeed, soy protein has been previously reported to regulate the expression of key transcription factors controlling lipid metabolism, such as SREBP-1, liver X receptor (LXR) and peroxisome proliferator activated receptor α (PPAR-α) [22]. These transcription factors control fatty acid desaturase 1 (*Fads1*) and 2 (*Fads2*) gene expression [23]. *Fads1* and *Fads2* genes encode the desaturase enzymes D5D and D6D, respectively [9,24]. Rats who were fed a soy protein diet for 160 days experienced a decrease in hepatic *Srebp-1c* mRNA expression, concomitant with a decrease in *Fads1* and *Fads2* gene expression, compared to rats fed a casein diet [25]. Thus, our results may stem from the transcriptional regulation of desaturase genes.

Another possible explanation for the differential effects of soy and casein consumption on the desaturation pathway may relate to differences in amino acid composition. Unlike casein, soy protein has a limited methionine content, an essential amino acid [26]. Sugiyama et al. [27] reported that the supplementation of a soy protein diet with L-methionine restored the microsomal AA/LA ratio to that observed in a casein-based diet. Further, Shimada et al. [28] found that the supplementation of a casein-based diet with L-methionine increased microsomal D6D activity compared to a control casein diet. We examined methionine intake using FFQ data in all subjects, but did not find a consistent pattern between the groups (in either our primary or secondary analyses) that aligned with differences in estimated D6D activity (data not shown); however, measuring methionine levels in circulation will provide greater clarity around this potential explanation for our findings.

The aforementioned mechanisms provide potential explanations for why LC-PUFA levels are lower in subjects who consume soy, irrespective of fluid milk and/or dairy food intake. Further elucidation using stable isotope fatty acid tracers to study LC-PUFA synthesis in subjects who consume soy beverages/foods or soy protein isolates will help clarify the relationship between soy consumption and fatty acid flux through the D6D/D5D pathway. Moreover, measuring hepatic desaturase mRNA and protein expression, as well as transcription factors known to regulate the desaturation pathway, will provide further mechanistic insights regarding the relationship between soy and D6D activity.

The design of the cross-sectional TNH study is potentially limited by the narrow age range of the participants, since individuals were between the ages of 20 and 29 years. Although our findings may not be generalizable to the entire population, these results set the stage for future research on fatty acid desaturation regulation by the consumption of soy foods, which should include individuals from different age groups. Another limitation of the cross-sectional study is the use of an FFQ to obtain dietary intake data from subjects, which are prone to inaccurate reporting. For example, food intake may be underestimated, especially if participants are not familiar with portion sizes and cooking measurements (e.g., cups, milliliters, or ounces). Furthermore, previous findings suggest that dietary recall declines quickly, and that the information provided by individuals generally comes from the short-term memories of their typical diet [29]. Nevertheless, FFQs are a validated tool that can be readily used in large-scale studies, such as the TNH study. The present study is also limited by using plasma fatty acids to estimate enzyme activities. This is notable since plasma fatty acids reflect both dietary intake and endogenous production. Except for ALA and LA (both essential fatty acids), it is not possible to definitively know the origin of other fatty acids measured in plasma without using tracers. Soy beverage contains more LA and ALA [30] than bovine milk [31]. Thus, the higher plasma ALA and LA levels found in individuals consuming soy beverage compared to milk/dairy may reflect differences in the essential fatty acid content of soy beverage and milk/dairy. Past research showed that a higher ALA and LA intake increases desaturation activity and that the LA/ALA ratio in the diet is important [13]. We accounted for LA and ALA plasma concentrations in our multivariate models, and findings from our primary and secondary analyses indicated that, despite individuals consuming soy having higher plasma ALA and LA, the estimated D6D activity using product-to-precursor ratios was lower compared to individuals consuming milk/dairy. While we acknowledge that product-to-precursor ratios are not a direct measure of enzymatic activity, a previous report suggests that these estimates generally align with those obtained using radiolabeled fatty acids [32]. As such, our results, in conjunction with past research in rodent models, suggest that increased soy intake suppresses D6D activity. Future clinical trials using labeled essential fatty acids are needed to definitively conclude whether D6D activity is suppressed with soy. Moreover, future studies are necessary to elucidate which component of soy (e.g., soy protein, lower methionine content, isoflavones, etc.) may be responsible for suppressing D6D activity.

## 5. Conclusions

In conclusion, we report an inverse association between soy intake and estimated desaturase activities and omega-6 LC-PUFA levels. With the growing popularity and availability of plant-based beverages, many consumers have moved away from consuming fluid milk. Indeed, soy beverage is a common plant-based alternative in the market, with a compound annual growth rate estimated at ~6% by 2025 [33]. Consequently, we anticipate that our findings may have ramifications for public health nutrition. Indeed, these findings are particularly important given that the Western diet is low in omega-3 LC-PUFA such as EPA and DHA, but high in omega-6 LC-PUFA [34]. Low levels of EPA and DHA in the body have been associated with an increased risk of non-alcoholic fatty liver disease, neuropsychiatric disorders, and cardiovascular disease, among other conditions [35,36,37]. As such, individuals consuming soy on a regular basis in the context of a low omega-3 LC-PUFA diet may benefit from increasing these important fatty acids in their diets to offset potential reductions in their endogenous production. These results highlight the importance of continued research regarding the role of soy in LC-PUFA synthesis.

## Figures and Tables

**Table 1:** General characteristics of the population by beverage intake subgroups ^1,2^.

Variables	Total Population (*n* = 1083)	Group “Milk” (*n* = 671)	Group “Soy” (*n* = 71)	Group “Both” (*n* = 269)	Group “No Consumption” (*n* = 72)	*p*-Value
Sex	F: 764 (70.5%) M: 319 (29.5%)	F: 466 (69.4%) M: 205 (30.6%)	F: 55 (77.5%) M: 16 (22.5%)	F: 195 (72.5%) M: 74 (27.5%)	F: 48 (66.7%) M: 24 (33.3%)	-
Ethnicity						
Caucasian	459	305	36	91	27	-
East Asian	396	196	27	145	28	-
South Asian	125	99	4	13	9	-
Other	103	70	4	20	8	-
Bioclinical markers						
Age (yrs)	22.7 ± 2.5	22.66 ± 2.47 ^a,b^	23.30 ± 2.76 ^a^	22.41 ± 2.32 ^b^	22.88 ± 2.64 ^a,b^	0.0468
BMI (kg/m^2^)	22.76 ± 3.49	23.03 ± 3.62 ^a^	22.11 ± 3.02 ^a,b^	22.24 ± 2.99 ^b^	22.78 ± 4.19 ^a,b^	0.0058
TAG (mmol/L)	0.94 ± 0.45	0.95 ± 0.47	0.91 ± 0.34	0.93 ± 0.44	0.95 ± 0.40	0.8052
hs-CRP (mg/L)	1.26 ± 2.68	1.42 ± 3.00	0.92 ± 1.46	0.99 ± 2.18	1.09 ± 2.06	0.0852
Total cholesterol (mmol/L)	4.23 ± 0.77	4.24 ± 0.79	4.17 ± 0.70	4.18 ± 0.73	4.39 ± 0.82	0.1738
LDL (mmol/L)	2.23 ± 0.64	2.25 ± 0.66	2.14 ± 0.55	2.18 ± 0.60	2.34 ± 0.70	0.1044
HDL (mmol/L)	1.57 ± 0.39	1.55 ± 0.39	1.61 ± 0.41	1.59 ± 0.38	1.62 ± 0.41	0.2331
Glucose (mmol/L)	4.78 ± 0.43	4.78 ± 0.37	4.83 ± 0.97	4.76 ± 0.35	4.83 ± 0.46	0.5653
Insulin (pmol/L)	48.48 ± 35.66	48.33 ± 28.90	43.35 ± 23.59	48.81 ± 49.10	53.68 ± 42.21	0.3906
Dietary intake (FFQ)						
Total kcal per day	1956 ± 647	1963 ± 637 ^a^	1877 ± 673 ^a,b^	2015 ± 659 ^a^	1748 ± 629 ^b^	0.0127
Omega-6/Omega-3 (g)	10.22 ± 15.52	9.83 ± 9.27 ^a^	15.07 ± 47.16 ^b^	10.14 ± 12.94 ^a,b^	9.30 ± 2.67 ^a,b^	0.0539
ALA and LA plasma concentrations (gas chromatography)						
LA (18:2*n*6) (μg/mL)	609.1 ± 168.7	596.4 ± 171.0 ^a^	663.7 ± 145.2 ^b^	622.7 ± 163.5 ^a,b^	622.3 ± 176.2 ^a,b^	0.0036
ALA (18:3*n*3) (μg/mL)	14.38 ± 7.22	13.90 ± 7.38	16.12 ± 6.30	15.00 ± 6.87	14.79 ± 7.54	0.0254
LA/ALA (μg/mL)	47.49 ± 15.57	48.34 ± 16.26	45.29 ± 14.75	45.59 ± 12.67	48.92 ± 18.74	0.0451

^1^ Results are expressed as means ± SDs. Differences between groups were determined using an ANCOVA and post hoc Tukey HSD test (JMP V14). Abbreviations: BMI, body mass index; TAG: triglycerides; hs-CRP: high sensitivity-C-reactive protein; LD: low-density lipoprotein; HDL: high-density lipoprotein. ^2^ Values in each row sharing the same superscript letter are not significantly different from one another.

**Table 2:** Plasma polyunsaturated fatty acid levels and ratios by beverage intake subgroups ^1,2^.

Fatty Acid	% Value					
	Total Population (*n* = 1083)	Group “Milk” (*n* = 671)	Group “Soy” (*n* = 71)	Group “Both” (*n* = 269)	Group “No Consumption” (*n* = 72)	*p*-Value ^5^
Plasma fatty acids ^3^						
ALA (18:3*n*-3)	0.75 ± 0.26	0.72 ± 0.26 ^a^	0.83 ± 0.27 ^b^	0.79 ± 0.22 ^b^	0.74 ± 0.33 ^a,b^	<0.0001 ^$^
EPA (20:5*n*-3)	0.61 ± 0.43	0.59 ± 0.38	0.54 ± 0.33	0.65 ± 0.54	0.68 ± 0.54	0.0643
DHA (22:6*n*-3)	1.56 ± 0.54	1.54 ± 0.51 ^a,b^	1.42 ± 0.54 ^b^	1.63 ± 0.58 ^a^	1.66 ± 0.59 ^a^	0.0047
LA (18:2*n*-6)	32.36 ± 4.08	31.81 ± 3.94 ^a^	34.17 ± 3.65 ^b^	33.42 ± 3.87 ^b^	31.78 ± 5.09 ^a^	<0.0001 ^$^
GLA (18:3*n*-6)	0.33 ± 0.16	0.34 ± 0.16 ^a^	0.31 ± 0.15 ^a,b^	0.30 ± 0.16 ^b^	0.32 ± 0.15 ^a,b^	0.0043
DGLA (20:3*n*-6)	1.23 ± 0.36	1.27 ± 0.37 ^a^	1.14 ± 0.30 ^b^	1.15 ± 0.33 ^b^	1.16 ± 0.37 ^b^	<0.0001 ^$^
AA (20:4*n*-6)	6.20 ± 1.40	6.35 ± 1.41 ^a^	5.90 ± 1.38 ^b^	5.87 ± 1.37 ^b^	6.25 ± 1.20 ^a,b^	<0.0001 ^$^
Product-to-precursor ratios ^4^						
AA/LA	0.20 ± 0.07	0.20 ± 0.08 ^a^	0.19 ± 0.05 ^a,b^	0.18 ± 0.05 ^b^	0.21 ± 0.12 ^a^	<0.0001 ^$^
EPA/ALA	0.89 ± 0.69	0.88 ± 0.63	0.88 ± 0.52	0.89 ± 0.83	0.90 ± 0.80	0.9766
D5D	5.48 ± 2.05	5.40 ± 2.04	5.72 ± 2.12	5.52 ± 1.86	5.96 ± 2.68	0.0862
D6D	0.0106 ± 0.0066	0.0111 ± 0.0062 ^a^	0.0099 ± 0.0053 ^a,b^	0.0093 ± 0.0057 ^b^	0.0116 ± 0.0123 ^a^	0.0004 ^$^
ELOVL2	0.0481 ± 0.0734	0.0493 ± 0.0747	0.0582 ± 0.0747	0.0444 ± 0.0693	0.0419 ± 0.0765	0.4262
ELOVL5	4.57 ± 2.85	4.50 ± 2.58	4.44 ± 2.38	4.90 ± 3.61	4.21 ± 2.30	0.1374

^1^ Results are expressed as means ± SDs. Differences between groups were determined using an ANCOVA and post hoc Tukey HSD test (JMP V14). Abbreviations: D5D: delta-5 desaturase; D6D: delta-6 desaturase; ELOVL2: Elongation of Very-Long-Chain Fatty Acids Protein 2; ELOVL5: Elongation of Very-Long-Chain Fatty Acids Protein 5. ^2^ Values in each row sharing the same superscript letter are not significantly different from one another. ^3^ Covariates included in the model for fatty acids: sex, BMI, age, waist circumference, ethnicity, total caloric intake, and omega-3 supplementation. ^4^ Covariates included in the model for product-to-precursor ratios: sex, BMI, age, waist circumference, ethnicity, total caloric intake, omega-3 supplementation, plasma ALA, LA and DHA levels (μg/mL). ^5^ The ^$^ symbol indicates results that remained significant after the Bonferroni correction for multiple testing (*p* = 0.05/13 tests = 0.004).

**Table 3:** General characteristics of the population by total dairy and total soy intake ^1^.

Variables	Total Population (*n* = 1064)	Group “Dairy” (*n* = 401)	Group “Both” (*n* = 663)	*p*-Value
Sex	F: 751 (70.6%) M: 313 (29.4%)	F: 267 (66.6%) M: 134 (33.4%)	F: 484 (73%) M: 179 (27%)	-
Ethnicity				
Caucasian	448	214	234	-
Asian	391	53	338	-
South Asian	124	76	48	-
Other	99	57	42	-
Bioclinical markers				
Age (yrs)	22.63 ± 2.45	22.60 ± 2.42	22.64 ± 2.47	0.7936
BMI (kg/m^2^)	22.79 ± 3.50	23.40 ± 3.79	22.42 ± 3.26	<0.0001
TAG (mmol/L)	0.94 ± 0.45	0.95 ± 0.48	0.94 ± 0.43	0.7270
hs-CRP (mg/L)	1.27 ± 2.71	1.52 ± 2.97	1.12 ± 2.53	0.0269
Total cholesterol (mmol/L)	4.23 ± 0.77	4.27 ± 0.78	4.21 ± 0.77	0.2070
LDL (mmol/L)	2.24 ± 0.64	2.30 ± 0.65	2.19 ± 0.63	0.0082
HDL (mmol/L)	1.57 ± 0.39	1.54 ± 0.39	1.59 ± 0.39	0.0192
Glucose (mmol/L)	4.78 ± 0.44	4.78 ± 0.37	4.78 ± 0.47	0.8169
Insulin (pmol/L)	48.63 ± 35.77	51.05 ± 32.27	47.17 ± 37.67	0.0756
Dietary intake (FFQ)				
Total kcal per day	1958 ± 644	1950 ± 669	1962 ± 628	0.7671
Omega-6/Omega-3 (g)	9.83 ± 9.85	9.68 ± 3.94	9.92 ± 12.10	0.6419
ALA and LA plasma concentrations (gas chromatography)				
LA (18:2*n*6) (μg/mL)	608.1 ± 168.9	583.9 ± 170.4	622.7 ± 166.5	0.0003
ALA (18:3*n*3) (μg/mL)	14.27 ± 7.09	13.50 ± 6.93	14.73 ± 7.15	0.0057
LA/ALA (μg/mL)	47.61 ± 15.49	48.99 ± 16.93	46.78 ± 14.50	0.0301

^1^ Results are expressed as means ± SDs. Differences between groups were determined using an ANCOVA and Student’s *t*-test (JMP V14). Abbreviations: BMI: body mass index; TAG: triglycerides; hs-CRP: high sensitivity-C-reactive protein; LDL: low-density lipoprotein; HDL: high-density lipoprotein.

**Table 4:** Plasma polyunsaturated fatty acids and ratios by total dairy and total soy intake ^1^.

Fatty Acid	% Value			
	Total Population (*n* = 1064)	Group “Dairy” (*n* = 401)	Group “Both” (*n* = 663)	*p*-Value ^4^
**Plasma fatty acids ^2^**				
ALA (18:3*n*-3)	0.74 ± 0.25	0.71 ± 0.28	0.76 ± 0.23	0.0009 ^$^
EPA (20:5*n*-3)	0.61 ± 0.43	0.60 ± 0.39	0.62 ± 0.45	0.4991
DHA (22:6*n*-3)	1.57 ± 0.52	1.49 ± 0.50	1.62 ± 0.54	<0.0001 ^$^
LA (18:2*n*-6)	32.32 ± 4.06	31.53 ± 3.98	32.80 ± 4.02	<0.0001 ^$^
GLA (18:3*n*-6)	0.33 ± 0.16	0.35 ± 0.17	0.31 ± 0.15	<0.0001 ^$^
DGLA (20:3*n*-6)	1.23 ± 0.36	1.30 ± 0.36	1.18 ± 0.35	<0.0001 ^$^
AA (20:4*n*-6)	6.20 ± 1.40	6.59 ± 1.41	5.98 ± 1.34	<0.0001 ^$^
**Product-to-precursor ratios ^3^**				
AA/LA	0.20 ± 0.07	0.21 ± 0.07	0.19 ± 0.07	<0.0001 ^$^
EPA/ALA	0.89 ± 0.68	0.93 ± 0.63	0.86 ± 0.71	0.0824
D5D	5.48 ± 2.02	5.45 ± 2.05	5.50 ± 2.01	0.7042
D6D	0.0106 ± 0.0066	0.0117 ± 0.0076	0.0099 ± 0.0058	<0.0001 ^$^
ELOVL2	0.0484 ± 0.0738	0.0500 ± 0.0705	0.0467 ± 0.0758	0.5918
ELOVL5	4.59 ± 2.86	4.45 ± 2.78	4.67 ± 2.90	0.2250

^1^ Results are expressed as means ± SDs. Differences between groups were determined using an ANCOVA and Student’s *t*-test (JMP V14). Abbreviations: D5D: delta-5 desaturase; D6D: delta-6 desaturase; ELOVL2: Elongation of Very-Long-Chain Fatty Acids Protein 2; ELOVL5: Elongation of Very-Long-Chain Fatty Acids Protein 5. ^2^ Covariates included in the model for fatty acids: sex, BMI, age, waist circumference, ethnicity, total caloric intake, and omega-3 supplementation. ^3^ Covariates included in the model for product-to-precursor ratios: sex, BMI, age, waist circumference, ethnicity, total caloric intake, omega-3 supplementation, plasma ALA, LA and DHA levels (μg/mL). ^4^ The ^$^ symbol indicates results that remained significant after Bonferroni correction for multiple testing (*p* = 0.05/13 tests = 0.004).

## Data Availability

Data is contained within the article.
